# Immunophenotyping of pulmonary sarcomatoid carcinoma

**DOI:** 10.3389/fimmu.2022.976739

**Published:** 2022-10-20

**Authors:** Yu Ma, Wensheng Li, Zhenzhen Li, Jie Chen, Hongtao Wang, Tao Jiang, Jianfei Zhu

**Affiliations:** ^1^ Department of Pathology, Shaanxi Provincial People’s Hospital, Xi’an, China; ^2^ Department of Thoracic Surgery, Shaanxi Provincial People’s Hospital, Xi’an, China; ^3^ Department of Thoracic Surgery, Tangdu Hospital, Fourth Military Medical University, Xi’an, China

**Keywords:** pulmonary sarcomatoid carcinoma, non-small cell lung cancer, immune checkpoint inhibitors, immunophenotype, T cell infiltration, PD-L1

## Abstract

**Background:**

Previous studies have suggested that patients with pulmonary sarcomatoid carcinoma (PSC)may benefit from immune checkpoint inhibitors (ICIs); however, relevant data are lacking. This study aimed to establish the immunophenotype of PSC by assessing PD-L1 and CD8+ T-cell infiltration.

**Methods:**

A retrospective analysis of pathologically confirmed PSC cases from two centers was performed from January 2009 to May 2021. According to the infiltration of CD8+ T cells in different spatial regions, patients were classified into three types: immune-inflamed, immune-excluded, and immune desert. PD-L1 staining was also performed on the intratumoral region and the tumor proportion score (TPS) was used for scoring. Combined with CD8+ T-cell infiltration and PD-L1 expression in the intratumoral region, immunophenotyping can be divided into four types: type I (PD-L1+/CD8+, adaptive immune resistance), type II (PD-L1-/CD8-, immunologic ignorance), type III (PD-L1+/CD8-, intrinsic induction), and type IV (PD-L1-/CD8+, tolerance). Finally, correlation analysis was performed on the immunophenotype, clinicopathological characteristics, and outcomes of the patients.

**Results:**

A total of 32 patients with PSC were included in the final analysis. Of these patients, 65.6% (21/32), 15.6% (5/32), and 18.8% (6/32) were classified as immune-inflamed, immune-excluded, and immune-desert, respectively. Notably, the immune-inflamed type is predominantly observed in pleomorphic carcinomas (PC, 66.7%). Moreover, among these participants, 19 (59.4%) were classified as PD-L1 positive according to the TPS score. In particular, 11 (34.4%) patients had PD-L1 TPS scores >50%. Next, we immunophenotyped patients with PSC based on CD8+ T cell infiltration and tumor cell PD-L1 expression (types I–IV). Type I (PD-L1+/CD8+, adaptive immune resistance) was the most prevalent subtype, accounting for 46.9% (15/32), followed by type II (PD-L1-/CD8-, immunological ignorance) (21.9%), type IV (PD-L1-/CD8+, tolerance) (18.7%), and type III (PD-L1+/CD8-, intrinsic induction) (12.5%). Finally, we performed a survival analysis and found that neither immunophenotype was a predictor of prognosis in patients with PSC. Multivariate analysis showed that pneumonectomy increased the risk of death by four times compared with lobectomy (RR: 4.1; 95% CI:1.3-12.4, *P*=0.014).

**Conclusion:**

Patients with PSC are characterized by immune-inflamed type and type I (PD-L1+/CD8+, adaptive immune resistance), explaining the intrinsic reasons for their high response rate to immunotherapy.

## Introduction

Pulmonary sarcomatoid carcinoma (PSC) is a rare, specialized subtype of non-small cell lung cancer (NSCLC) with biphasic differentiation between carcinoma and sarcoma, accounting for 0.1-0.4% of all lung malignancies (1, 2). Based on the fourth edition of the WHO classification of lung tumors, PSC can be divided into five subtypes: pleomorphic carcinoma (PC), spindle cell carcinoma (SCC), giant cell carcinoma (GCC), carcinosarcoma (CaS), and pulmonary blastoma (PB) (3). Its subclassification has not been changed in the latest fifth edition (4). Heterogeneous from other types of NSCLC, PSC biological behavior is more aggressive, coupled with its resistance to traditional treatment modalities, with an overall five-year survival rate of less than 20% (5–7). Therefore, there is an urgent need to identify novel treatment modalities for PSC.

Immune checkpoint inhibitors (ICIs) bring about the possibility of long-term survival in patients with NSCLC, and immunotherapy is a milestone development for patients with NSCLC (8, 9). The efficacy of ICIs in PSC lacks large data reports, but some scattered data still show the superiority of ICIs in the treatment of PSC (10–13). In the largest relevant study to date, although the sample size was only 38 patients, 54% of patients in this study received second-line nivolumab therapy, and 46% received third-line or more therapy. Regardless of PD-L1 status, the objective response rate (ORR) was 40.5% and the disease control rate (DCR) was 64.8%, suggesting that patients with PSC have a higher response rate and longer overall survival (OS) after ICI treatment (14). The underlying reason for this has always been a research hotspot. PD-L1, a biomarker for predicting the efficacy of ICI, was confirmed to be more frequently expressed in PSC than in other common subtypes of NSCLC (15). In addition, Kotlowska et al. reported that there was more immune and inflammatory cell infiltration in PSC, thus defining PSC as “hot tumors” (16). Recent studies have confirmed that immunophenotyping based on CD8+ T cell infiltration and PD-L1 expression in the intratumoral region is a better choice for predicting the efficacy of NSCLC immunotherapy, but there are no related reports on PSC (17).

To bridge this gap, we conducted a large, retrospective study. In the present study, CD8+ T cell infiltration was assessed in different spatial regions of the intratumoral and peritumoral regions, and PD-L1 expression in the intratumoral region was detected. This study aimed to establish immunophenotyping based on CD8+ T cell infiltration in different regions, and on combined CD8+ T cell infiltration and PD-L1 expression, and to analyze the relationship between immunophenotyping and the clinicopathological characteristics of patients with PSC. These results may provide support for understanding the tumor microenvironment (TME) of PSCs and their personalized immune therapy.

## Methods

### Study design

**Figure 1:**
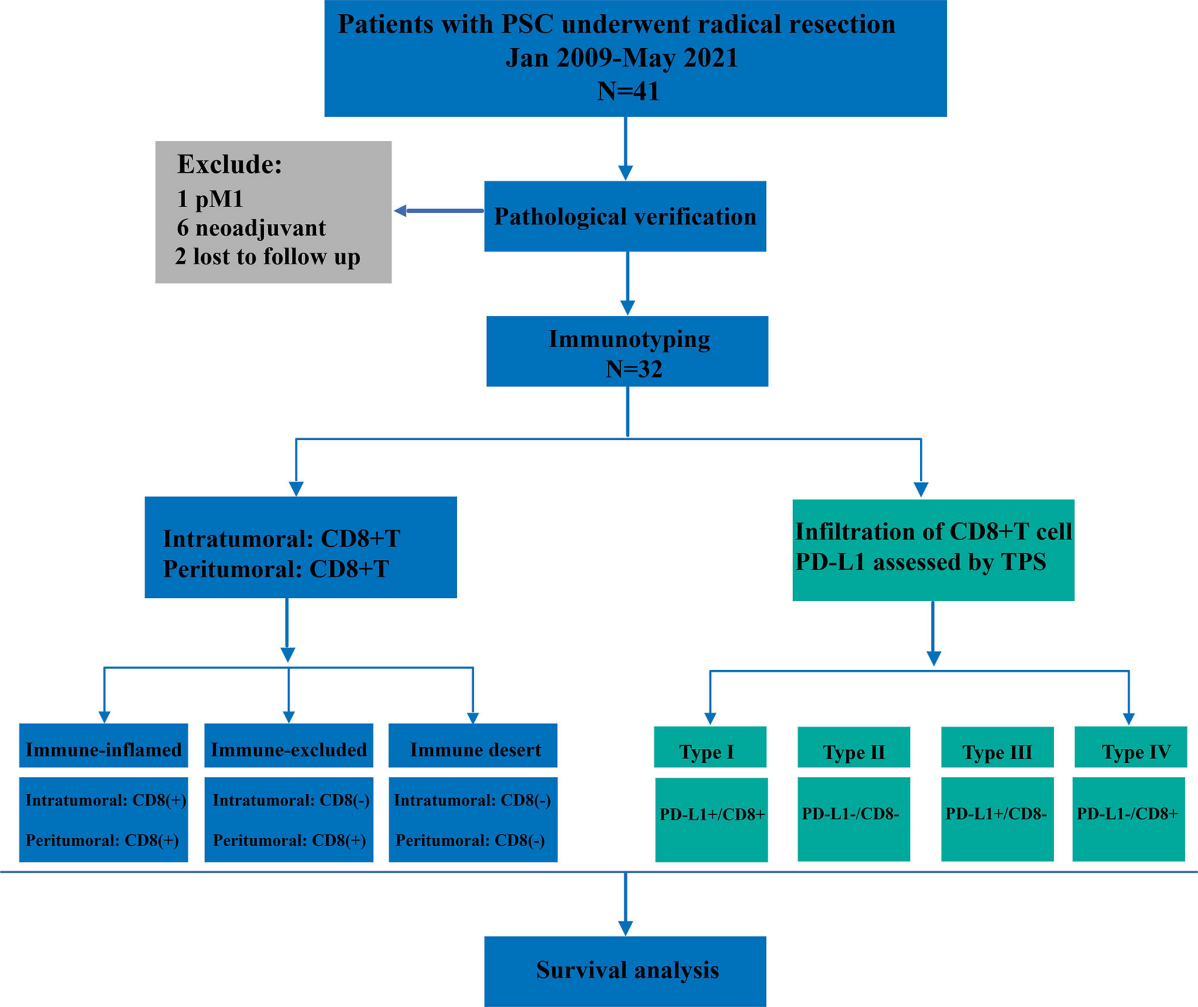
A flow chart of the research process.

For the final analysis, we retrospectively enrolled patients with PSC who underwent radical resection from January 2009 to May 2021 (**Figure 1**). All patients included in this study were from two centers of thoracic surgery, namely Shaanxi Provincial People’s Hospital and Tangdu Hospital of the Fourth Military Medical University. The inclusion criteria were as follows: (1) pathological diagnosis of NSCLC, (2) immunohistochemical (IHC) diagnosis of PSC, (3) R0 resection, (4) both cancerous and paracancerous tissue, and (5) M0 stage. All patients who met the following criteria were excluded from this study: (1) pulmonary sarcoma, (2) metastatic PSC, (3) previous history of other malignant tumors, (4) neoadjuvant therapy, and (5) perioperative death. This study was approved by the Ethics Committee of Shaanxi Provincial People’s Hospital (approval number:20220621) and all patients provided signed informed consent.

### Diagnosis and subtyping of PSC

**Figure 2:**
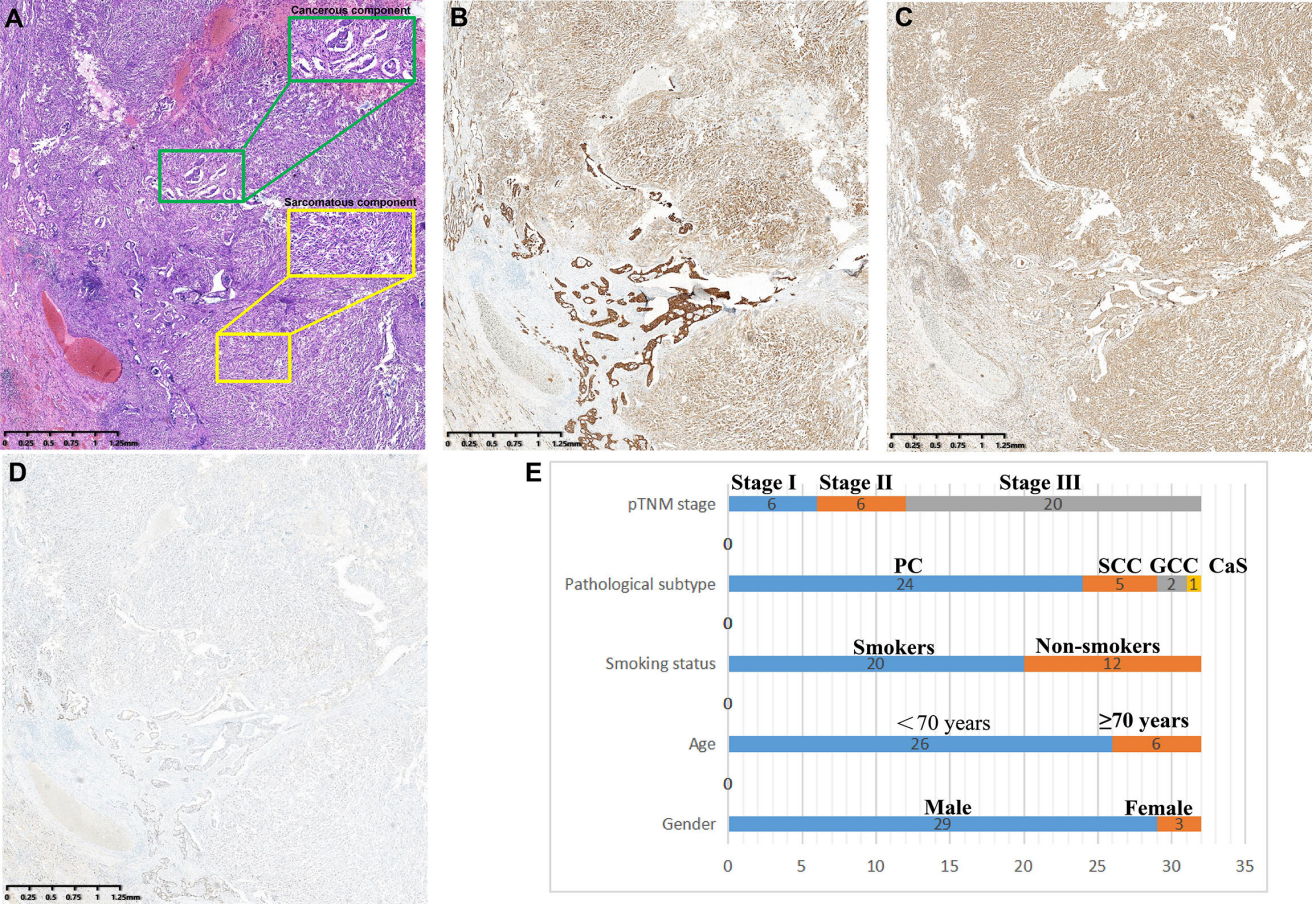
Clinicopathological features of PSCs. **(A)** Spindle cells with atypia mixed with adenocarcinoma components. **(B)** pan-CK was positive in both sarcoma and carcinoma components. **(C)** Vimentin is specifically expressed in sarcoma components but not in cancerous components. **(D)** TTF-1 was strongly positive in the adenocarcinoma component and weakly positive in the sarcoma component. **(E)** Subtype distribution of PSCs.

PSC is defined as NSCLC with both sarcomatous and carcinomatous components, and must be diagnosed by combined HE and IHC on surgically resected specimens. All diagnoses were made by three independent experienced pathologists according to the WHO (5^th^ edition) lung tumor classification criteria (4), and a fourth pathologist checked for inconsistency. PSC can be divided into five subtypes: PC, SCC, GCC, CaS, and PB. (1) PC refers to a carcinoma that contains at least 10% or more spindle cells or giant cell components in adenocarcinoma, squamous cell carcinoma, or undifferentiated NSCLC; undifferentiated carcinomas composed entirely of spindle or giant cells (including multinucleated cells) were defined as (2) SCC or (3) GCC, respectively; (4) CaS refers to a cancer that is mixed with heterologous sarcoma components (such as rhabdomyosarcoma, osteosarcoma, chondrosarcoma, etc.) in typical lung squamous cell carcinoma or adenocarcinoma; and (5) PB is a bidirectional malignant tumor composed of low-grade foetal adenocarcinoma and primitive mesenchymal components (**Figure 2**).

### Immunophenotyping of PSC

Two consecutive 4-micron thick sections from each patient were stained with monoclonal antibodies against CD8 (SP16) and PD-L1 (SP263) to assess the infiltration density of CD8+ T cells and the expression level of PD-L1, respectively. Immunohistochemical staining was performed using an automated immunostainer and an Ultra View Universal DAB (3,3’-diaminobenzidine) detection kit (Ventana Medical Systems, Inc. Tucson, AZ, USA). The same method was used for CD8 staining of paired paracancerous tissue. Placenta and tonsil tissues were respectively used as control for PD-L1 and CD8. All microscopic analyzes were performed under a light microscope (Zeiss, Germany).

The density of CD8+ T cell infiltration was defined as the proportion of CD8+T cells in nucleated cells in the stromal component of each specimen, and a proportion ≥5% was defined as positive for CD8+ T cell infiltration, and vice versa (18). Next, we performed immunophenotyping based on CD8+ T cell infiltration in PSC patients and defined the intratumoral region as tumor parenchyma and the peritumoral region as tumor cell nests around the stroma according to the previous definition criteria (19, 20). According to the infiltration of CD8+ T cells in different regions, the patients were divided into immune-inflamed, immune-excluded, and immune-desert types. Immune inflammation was defined as positive infiltration of CD8+ T cells in both the intratumoral and peritumoral regions; immune desert was defined as negative CD8+ T infiltration in both regions. The immune excluded was defined as the intratumoral region being negative for CD8+T infiltration and the peritumoral region being positive for CD8+ T infiltration (21).

The expression of PD-L1 was assessed according to the tumor proportion score (TPS) system recommended by clinical guidelines, and positive PD-L1 expression was defined as >1% of all tumor cells with positive tumor cell membranes in a given sample (22). Immunophenotyping is divided into four types according to PD-L1 expression and CD8+ T cell infiltration in the tumor region: Type I (PD-L1+/CD8+, adaptive immune resistance), Type II (PD-L1-/CD8-, immunologic ignorance), Type III (PD-L1+/CD8-, intrinsic induction), and Type IV (PD-L1-/CD8+, tolerance) (23).

### Statistical analysis

Correlations between immunophenotyping and clinicopathological features were analyzed using the Chi-square test. Disease-free survival (DFS) and OS were calculated using the Kaplan-Meier method, and the log-rank test was used to assess differences in survival between groups. Statistically significant prognostic factors screened in the univariate analysis or clinically recognized variables affecting the prognosis of patients with PSC were included in the multivariate Cox regression analysis. A two-tailed test with a *P*-value of <0.05 was considered statistically significant. The above statistical analysis was performed using SPSS 22.0 and Stata 14.0.

## Results

### Clinicopathological features

A total of 32 patients with PSC were included in this study, and the clinicopathological characteristics of these participants are summarized in **Table 1**. The mean age of participants was 61.0 years (range:39.0-86.0 years). More than 90.0% of the participants were men. Approximately 62.5% of patients had a history of smoking. According to histological subtype, PC was the most common histological subtype (75.0%), followed by SCC (15.6%), GCC (6.3%) and CaS (3.1%). Notably, patients with PB were not included in the present study. Approximately 62.5% of patients had been diagnosed with stage III disease at their initial visit. Lobectomy was the predominant surgical procedure in this cohort, with a percentage as high as 81.3% (26/32); notably, the remaining 18.7% (6/32) of the patients underwent pneumonectomy.

**Table 1:** Clinicopathological characteristics of patients with PSC (N=32).

Variable	N	Percentage (%)
Age (61.0±10.23 )	Range: 39.0-86.0 years	
Gender		
Male	29	90.6%
Female	3	9.4%
Smoking status		
Non-smokers	12	37.5%
Smokers	20	62.5%
Pathological subtype		
Pleomorphic carcinoma	24	75.0%
Spindle-cell carcinoma	5	15.6%
Giant-cell carcinoma	2	6.3%
Carcinosarcoma	1	3.1%
pT stage		
T1+T2	8	25.0%
T3+T4	24	75.0%
pN stage		
N0	17	53.1%
N1	4	12.5%
N2	11	34.4%
pTNM stage		
Stage I	6	18.8%
Stage II	6	18.8%
Stage III	20	62.4%
Surgical approach		
Lobectomy	26	81.3%
Pneumonectomy	6	18.7%

### CD8+ T cell infiltration in PSC

**Figure 3:**
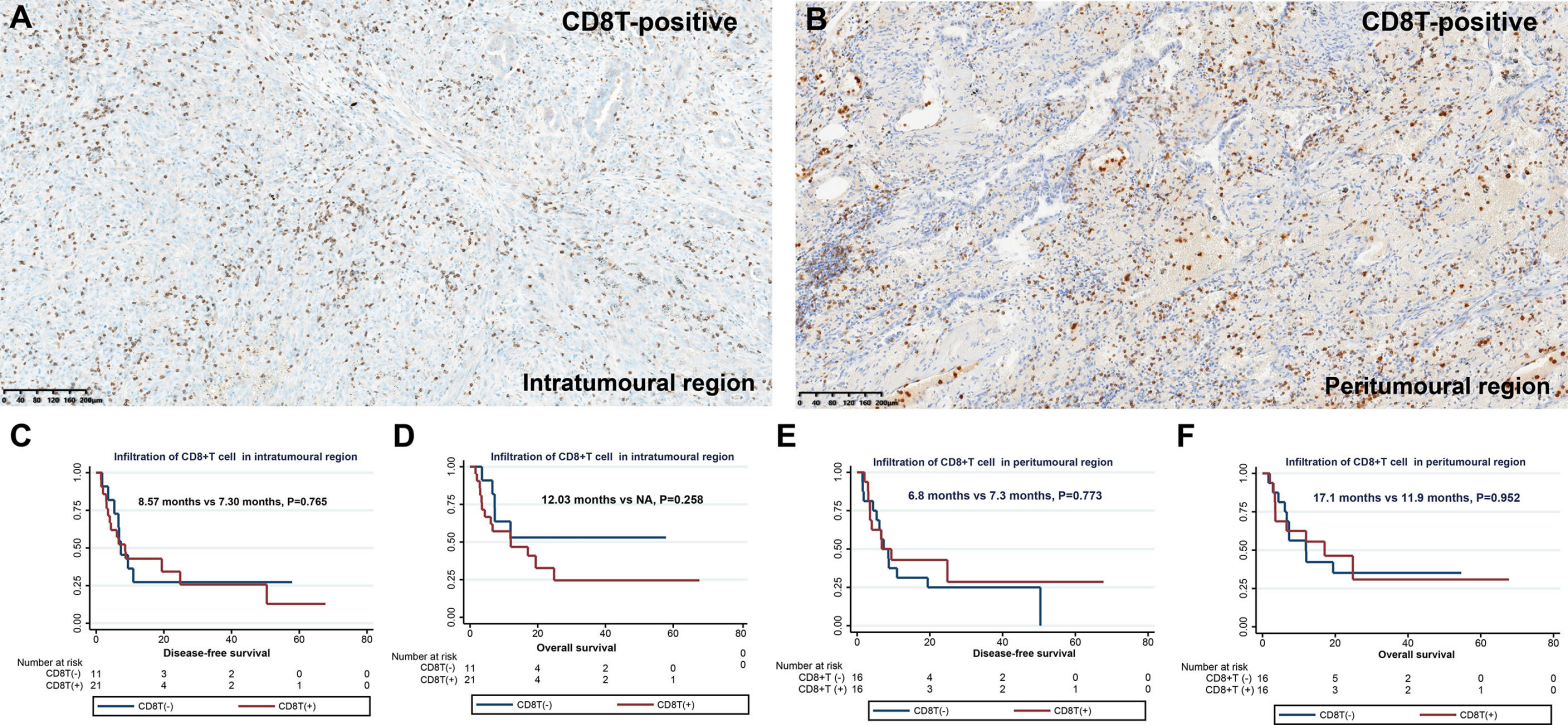
Infiltration and clinical outcome of CD8+ T cells in PSC. Infiltration of CD8+ T cells in the intratumoral region **(A)** and in the peritumoral region **(B)**; DFS **(C)** and OS **(D)** of different CD8+ T cell infiltration states in intratumoral region; DFS **(E)** and OS **(F)** of different CD8+ T cell infiltration states in peritumoral region.

Next, we observed the infiltration of CD8+ T cells in different spatial regions (intratumoral and peritumoral regions) of PSC (**Table 2** and **Figures 3A, B**). In the intratumoral region, the positive rate of CD8+ T cell infiltration (65.6%) was higher than that in the peritumoral region (50.0%), although the difference was not statistically significant (*P*>0.05). CD8+ T-cell infiltration was more common in SCC (80.0%), followed by PC (66.7%), and the proportion of CD8+ T-cell infiltration positive in CaS and GCC was comparable (**Figure 3C**). We also found no correlation between CD8+ T cell infiltration in the intratumoral region and pT (*P*>0.05), pN (*P*>0.05), and pTNM stages (*P*>0.05) in patients with PSC. Survival analysis showed that there was no difference in DFS and OS between the two groups of patients with or without CD8+ cell infiltration in the intratumoral region (**Figures 3C, D**), the same results were found in the peritumoral region (**Figures 3E, F**).

**Table 2:** Correlation between CD8+ T cell infiltration and clinicopathological features in PSC.

Variable	N	Intratumoural region	P value	Peritumouralregion	*P* value
Gender			0.534		1.000
Male	29	18 (62.1%)		14 (48.3%)	
Female	3	3 (100%)		2 (66.7%)	
Age			1.000		0.654
<70 years	26	17 (65.4%)		14 (53.8%)	
≥70 years	6	4 (66.7%)		2 (33.3%)	
Smoking status			0.250		
Non-smokers	12	6 (50.0%)		6 (50.0%)	1.000
Smokers	20	15 (75.0%)		10 (50.0%)	
Pathological subtype			0.458		0.276
Pleomorphic carcinoma	24	16 (66.7%)		14 (58.3%)	
Spindle-cell carcinoma	5	4 (80.0%)		2 (40.0%)	
Giant-cell carcinoma	2	1 (50.0%)		0 (0)	
Carcinosarcoma	1	0 (0)		0 (0)	
pT stage			0.397		0.685
T1+T2	8	4 (50.0%)		5 (62.5%)	
T3+T4	24	17 (70.8%)		11 (64.7%)	
pN stage			0.292		0.563
N0	17	10 (58.8%)		8 (47.1)	
N1	4	4 (100%)		3 (75.0%)	
N2	11	7 (63.6%)		5 (45.5%)	
pTNM stage			0.354		0.648
Stage I	6	3 (50.0%)		3 (50.0%)	
Stage II	6	3 (50.0%)		4 (66.7%)	
Stage III	20	15 (75.0%)		9 (45.0%)	

### PD-L1 expression in PSC

**Figure 4:**
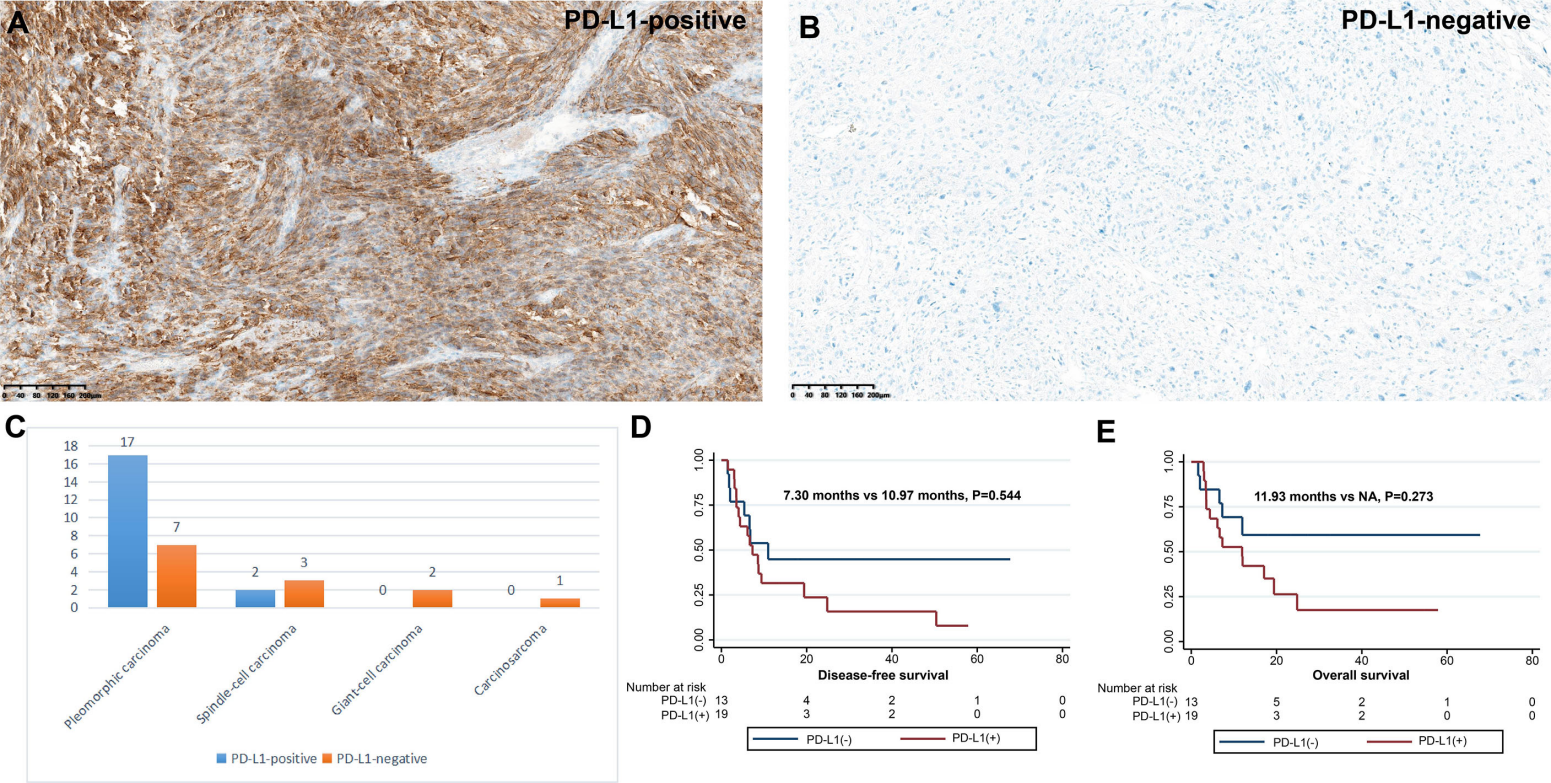
Expression and clinical outcome of PD-L1 in PSC. Criteria for positive **(A)** and negative **(B)** PD-L1 expression; **(C)** Infiltration density of PD-L1 expression in four subtypes of PSC. DFS **(D)** and OS **(E)** of different PD-L1 expression status.

Of the 32 participants, 19 (59.4%) were classified as PD-L1 positive according to the TPS score (**Table 3** and **Figure 4**). In particular, 11 (34.4%) patients had PD-L1 TPS scores>50%. PD-L1 positivity was the most common in PC (70.8%), followed by SCC (40.0%). In our analysis, PD-L1 expression was not associated with the pT stage of the tumor (*P*>0.05), and PD-L1 expression was not associated with lymph node metastasis (pN stage) (*P*>0.05). Notably, patients with positive CD8+ T-cell infiltration in the intratumoral region had higher PD-L1 expression than those with negative CD8+ T-cell infiltration (71.4% vs. 36.4%, *P*=0.072). Consistent with a previous study (15), as shown in **Figure 4**, PD-L1-positive PSC patients had a tendency to separate survival curves from PD-L1-negative patients, although the difference was not statistically significant (*P*=0.544).

**Table 3:** Correlation between PD-L1 expression and clinicopathological features in PSC.

Variable	N	PD-L1			*P* value
		Positive		Negative	
Gender					0.253
Male	29	16 (55.2%)		13 (44.8%)	
Female	3	3 (100%)		0 (0)	
Age					0.666
<70 years	26	16 (61.5%)		10 (38.5%)	
≥70 years	6	3 (50.0%)		3 (50.0%)	
Smoking status					0.150
Non-smokers	12	5 (41.7%)		7 (58.3%)	
Smokers	20	14 (70.0%)		6 (30.0%)	
Pathological subtype					0.091
Pleomorphic carcinoma	24	17 (70.8%)		7 (29.2%)	
Spindle-cell carcinoma	5	2 (40.0%)		3 (60.0%)	
Giant-cell carcinoma	2	0 (0)		2 (100%)	
Carcinosarcoma	1	0 (0)		1 (100%)	
pT stage					0.420
T1+T2	8	6 (75.0%)		2 (25.0%)	
T3+T4	24	13 (54.2%)		11 (45.8%)	
pN stage					0.384
N0	17	12 (70.6%)		5 (29.4%)	
N1	4	2 (50.0%)		2 (50.0%)	
N2	11	5 (45.5%)		6 (54.5%)	
pTNM stage					0.210
Stage I	6	5 (83.3%)		1 (16.7%)	
Stage II	6	2 (33.3%)		4 (66.7%)	
Stage III	20	12 (60.0%)		8 (40.0%)	
CD8+T cell infiltration					0.072
Positive	21	15 (71.4%)		6 (28.6%)	
Negative	11	4 (36.4%)		7 (63.6%)	

### Immunophenotyping of PSC based on CD8+ T cell infiltration

**Figure 5:**
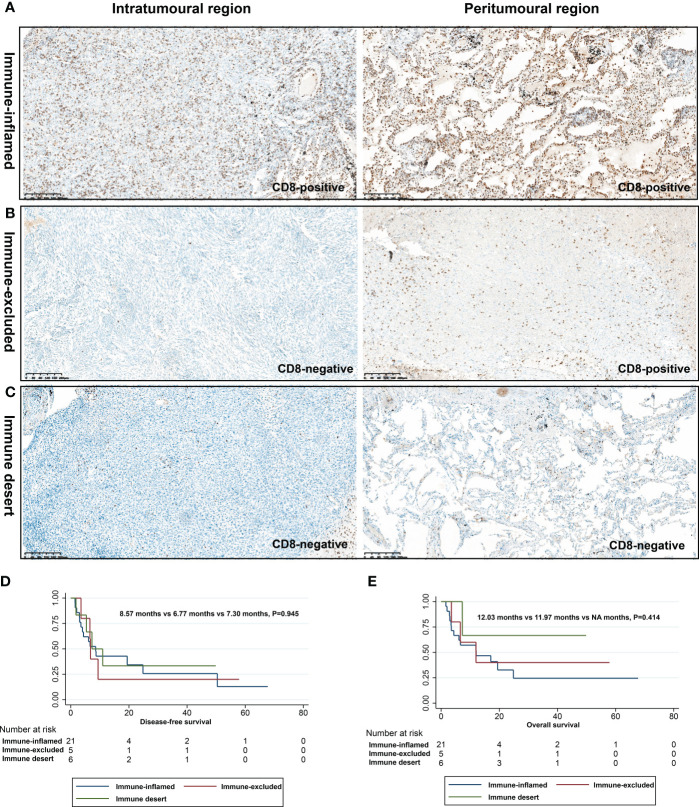
Immunophenotyping based on CD8+ T cell infiltration in different spatial regions. Immune inflamed type **(A)**, immune excluded type **(B)**, and immune desert type **(C)**; DFS **(D)** and OS **(E)** of different immunophenotypes.

To explore the response of PSC to immunotherapy, we investigated their immunophenotyping based on the infiltration of CD8+ T cells in different spatial regions, as shown in **Table 4** and **Figure 5**. Of these patients, 65.6% (21/32), 15.6% (5/32), and 18.8% (6/32) were classified as immune-inflamed, immune-excluded, and immune desert, respectively. Immune inflammation, a type of NSCLC immunophenotyping, is considered a predictor of response to immunotherapy in patients with NSCLC. In 21 cases of PSC recognized as immune-inflamed type, we found that the proportion of immune-inflamed type in PC and SCC was 66.7% and 80%, respectively. One patient with GCC and one CaS were classified as immune desert type. Next, we performed a correlation analysis between immunophenotyping and the clinicopathological features of PSC. The proportion of immune-inflamed type in stage I, II, and III patients was similar (50.0%, 50.0%, and 75.0%, respectively; *P* =0.329) (**Figures 5D, E**).

**Table 4:** Immunophenotyping of PSC based on CD8+ T cell infiltration.

Variable	N	Immunophenotype			*P* value
		Immune -inflamed	Immune-excluded	Immune desert	
Gender					0.420
Male	29	18 (62.1%)	5 (17.2%)	6 (20.7%)	
Female	3	3 (100%)	0 (0)	0 (0)	
Age					0.369
<70 years	26	17 (65.4%)	5 (19.2%)	4 (15.4%)	
≥70 years	6	4 (66.7%)	0 (0)	2 (33.3%)	
Smoking status					0.234
Non-smokers	12	6 (50.0%)	2 (16.7%)	4 (33.3%)	
Smokers	20	15 (75.0%)	3 (15.0%)	2 (10.0%)	
Pathological subtype					0.267
Pleomorphic carcinoma	24	16 (66.7%)	5 (20.8%)	3 (12.5%)	
Spindle-cell carcinoma	5	4 (80.0%)	0 (0)	1 (25.0%)	
Giant-cell carcinoma	2	1 (50.0%)	0 (0)	1 (50.0%)	
Carcinosarcoma	1	0 (0)	0 (0)	1 (100.0%)	
pT stage					0.143
T1+T2	8	4 (50.0%)	3 (37.5%)	1 (12.5%)	
T3+T4	24	17 (70.8%)	2 (8.3%)	5 (20.9%)	
pN stage					0.457
N0	17	10 (58.8%)	4 (23.5%)	3 (17.7%)	
N1	4	4 (100%)	0 (0)	0 (0)	
N2	11	7 (63.6%)	1 (9.1%)	3 (27.3%)	
pTNM stage					0.329
Stage I	6	3 (50.0%)	2 (33.3%)	1 (16.7%)	
Stage II	6	3 (50.0%)	2 (33.3%)	1 (16.7%)	
Stage III	20	15 (75.0%)	1 (5.0%)	4 (20.0%)	

### Immunophenotyping of PSC by CD8+ T cell infiltration and PD-L1 expression

To develop a better predictive model for predicting the efficacy of immunotherapy in PSC, the immune microenvironment was classified into four types (types I-IV) according to intratumoral PD-L1 expression and CD8+ T-cell infiltration. Among the 32 patients, type I (PD-L1+/CD8+, adaptive immune resistance) was the most prevalent subtype, accounting for 46.9% (15/32), followed by type II (PD-L1-/CD8-, immunological ignorance) (21.9%), type IV (PD-L1-/CD8+, tolerance) (18.7%), and type III (PD-L1+/CD8-, intrinsic induction) (12.5%). We analyzed the relationship between immunophenotype and pTNM stage, and although there was no statistical difference (*P*=0.338), the proportion of patients with immunophenotype I (55.0%) in stage III was higher than that in stage II (16.7%) and stage I (50.0%) (**Table 5**).

**Table 5:** Immunophenotyping of PSC based on CD8+ T cell infiltration and PD-L1 expression.

Variable	N	Immunophenotype				*P* value
		Type I	Type II	Type III	Type IV	
Gender						0.290
Male	29	12 (41.4%)	7 (26.9%)	4 (13.8%)	6 (17.9%)	
Female	3	3 (100%)	0 (0)	0 (0)	0 (0)	
Age						0.706
<70 years	26	12 (46.2%)	5 (19.2%)	4 (15.4%)	5 (19.2%)	
≥70 years	6	3 (50.0%)	2 (33.3%)	0 (0)	1 (16.7%)	
Smoking status						0.212
Non-smokers	12	4 (33.3%)	5 (41.7%)	1 (8.3%)	2 (16.7%)	
Smokers	20	11 (55.0%)	2 (10.0%)	3 (15.0%)	4 (20.0%)	
Pathological subtype						0.366
Pleomorphic carcinoma	24	13 (54.2%)	4 (16.7%)	4 (16.7%)	3 (12.4%)	
Spindle-cell carcinoma	5	2 (40.0%)	1 (20.0%)	0 (0)	2 (40.0%)	
Giant-cell carcinoma	2	0 (0)	1 (50.0%)	0 (0)	1 (50.0%)	
Carcinosarcoma	1	0 (0)	1 (100%)	0 (0)	0 (0)	
pT stage						0.334
T1+T2	8	4 (50.0%)	2 (25.0%)	2 (25.0%)	0 (0)	
T3+T4	24	11 (45.8%)	5 (20.8%)	2 (8.3%)	6 (25.1%)	
pN stage						0.228
N0	17	8 (47.1%)	3 (17.6%)	4 (23.5%)	2 (11.8%)	
N1	4	2 (50.0%)	0 (0)	0 (0)	2 (50.0%)	
N2	11	5 (45.5%)	4 (36.4%)	0 (0)	2 (18.1%)	
pTNM stage						0.338
Stage I	6	3 (50.0%)	1 (16.7%)	2 (33.3%)	0 (0)	
Stage II	6	1 (16.7%)	2 (33.3%)	1 (16.7%)	2 (33.3%)	
Stage III	20	11 (55.0%)	4 (20.0%)	1 (5.0%)	4 (20.0%)	

### Immunophenotyping of PSC and clinical outcomes

**Figure 6:**
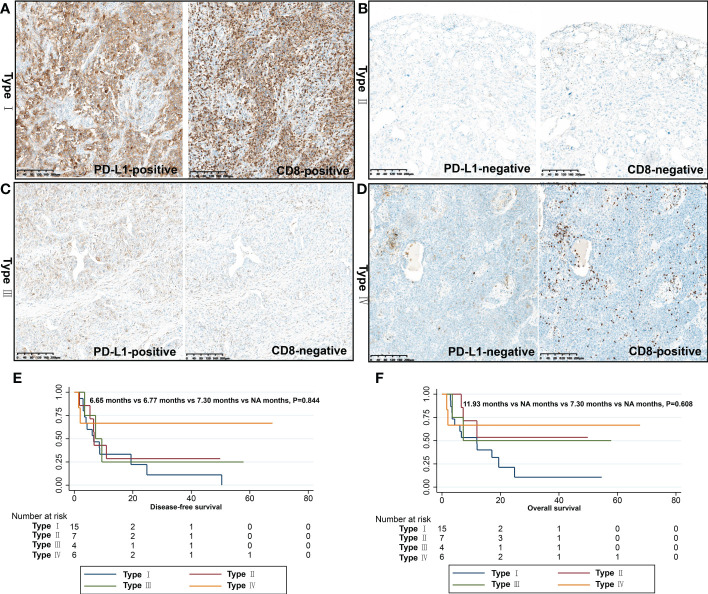
Immunophenotyping based on CD8+ T cell infiltration and PD-L1 expression. **(A)** Type I (PD-L1+/CD8+, adaptive immune resistance); **(B)** Type II (PD-L1-/CD8-, immunologic ignorance); **(C)** Type III (PD-L1+/CD8-, intrinsic induction); **(D)** Type IV (PD-L1-/CD8+, tolerance); DFS **(E)** and OS **(F)** of different immunophenotypes.

We performed survival analysis of patients with PSC, and the survival data are summarized in **Table 5**. In the present study, the median DFS and OS were 7.3 months and 12.0 months, respectively. In the univariate analysis, variables such as gender, age, smoking status, and pathological subtype did not affect DFS and OS in patients with PSC (*P*>0.05). Notably, the pN stage was the only variable that affected DFS in patients with PSC (*P*=0.029). In the OS analysis, we found that surgical approach (*P*=0.018) was only factor affecting the OS of these patients, and neither immunophenotype was a predictor of prognosis in patients with PSC (**Figures 5**, **6**).

Next, we found that neither immunophenotyping 1 based on the infiltration of CD8+ T cells in different regions nor immunophenotyping 2 based on the expression of CD8+ T cells and PD-L1 in the intratumoral region were prognostic indicators for patients with PSC (**Table 6**).

**Table 6:** Univariate analysis of DFS and OS of PSC.

Variable	DFS	95% CI	*P* value	OS	95% CI	*P* value
Gender			0.818			0.574
Male	7.30	3.70-10.90		17.07	4.49-29.64	
Female	8.57	0-17.48		11.93	0-26.23	
Age			0.710			0.725
<70 years	7.30	3.94-10.66		11.97	0-29.18	
≥70 years	5.37	0-10.81		17.07	6.36-27.78	
Smoking status			0.248			0.104
Non-smokers	9.40	0-33.69		NA	NA	
Smokers	6.65	5.26-8.05		7.3	0-18.95	
Surgical approach			0.094			0.018
Lobectomy	8.70	3.66-13.74		19.40	4.00-34.80	
Pneumonectomy	3.50	0-7.82		3.5	0-7.82	
Pathological subtype			0.912			0.842
Pleomorphic carcinoma	6.77	4.25-9.29		NA	NA	
Spindle-cell carcinoma	10.97	7.22-14.71		NA	NA	
Giant-cell carcinoma	1.80	NA		NA	NA	
Carcinosarcoma	5.37	NA		NA	NA	
pT stage			0.636			0.734
T1+T2	8.57	2.75-14.39		11.97	3.10-20.84	
T3+T4	6.65	3.57-9.74		12.03	0-25.10	
pN stage			0.029			0.151
N0	8.70	6.01-11.40		17.07	7.91-26.22	
N1	NA	NA		NA	NA	
N2	3.07	0-6.66		7.27	0-16.20	
pTNM stage			0.073			0.087
Stage I	10.97	0.57-21.37		19.40	4.77-34.04	
Stage II	9.40	8.03-10.77		NA	NA	
Stage III	5.37	1.50-9.23		6.65	5.19-8.11	
Adjuvant therapy			0.224			0.122
With	9.40	0-25.17		6.65	5.28-8.02	
Without	6.60	5.58-7.62		19.40	NA	
CD8+T cell infiltration			0.765			0.258
Positive	8.57	4.72-12.41		12.03	0-25.94	
Negative	7.30	4.28-10.32		NA	NA	
PD-L1 expression			0.544			0.273
Positive	7.30	3.84-10.77		11.93	4.28-19.59	
Negative	10.97	3.71-18.23		NA	NA	
Immunophenotype 1			0.945			0.414
Immune-inflamed	8.57	4.72-12.41		12.03	0-25.94	
Immune-excluded	6.77	6.41-7.13		11.97	0.44-23.49	
Immune desert	7.30	0.58-14.02		NA	NA	
Immunophenotype 2			0.844			0.608
Type I	6.65	1.35-11.95		11.93	4.48-19.39	
Type II	6.77	6.34-7.19		NA	NA	
Type III	7.30	1.55-13.05		7.30	NA	
Type IV	NA	NA		NA	NA	

Finally, we incorporated the four variables pT stage, pN stage, surgical approach, and PD-L1 expression into the Cox risk regression model for multivariate analysis (**Table 7**). The results showed that pneumonectomy increased the risk of death by four times compared to lobectomy (RR: 4.1, 95% CI:1.3-12.4, *P*=0.014). Notably, although not statistically different, PD-L1 expression in the intratumoral region increased the risk of recurrence/metastases (RR: 2.0, 95% CI: 0.8-5.2, *P*=0.136) and mortality (RR: 3.0, 95% CI: 1,0-8.8, *P*=0.053).

**Table 7:** Multivariate analysis of DFS and OS in patients with PSC.

Variable	DFS			OS		
	RR	95% CI	*P* value	RR	95% CI	*P* value
PD-L1 expression: positive/negative	2.0	0.8-5.2	0.136	3.0	1.0-8.8	0.053
Surgical approach: pneumonectomy/lobectomy	2.6	0.9-7.7	0.074	4.1	1.3-12.4	0.014
pT stage: T3+T4/T1+T2	0.9	0.3-2.7	0.953	0.9	0.3-2.6	0.802
pN stage: N1+N2+N3/N0	1.4	0.6-3.6	0.447	1.6	0.6-4.4	0.353

## Discussion

PSC, a deadly subtype of NSCLC with both carcinomatous and sarcomatous components, is the most aggressive form of NSCLC. Heterogeneous to the two most common subtypes of lung adenocarcinoma and squamous cell carcinoma in NSCLC, driver mutations, primarily KRAS and MET mutations, may be intrinsic drivers of the malignant features of PSC (24–26). Based on the above data, in clinical practice, the therapeutic effect of traditional platinum-based chemotherapy and targeted therapy on PSC is unsatisfactory (27, 28). Some scattered case reports confirm that ICI may be the dawn of PSC treatment, but there is a lack of relevant data to support it. This retrospective study was based on 32 patients with PSC from two centers. Immunophenotyping of PSC was established for the first time by detecting CD8+ T cell infiltration in the intratumoral and peritumoral regions, and PD-L1 expression in the intratumoral region.

Even in earlier TNM stage PSC, the prognosis is still poor. A study of 7965 patients with PSC from the US National Cancer Centre was included in the analysis (29), accounting for 0.5% of all NSCLC (7965/1,547,531). For patients with operable PSC, survival analysis showed a median OS of only 16.9 months for stage I-II patients and 5.8 months for stage III patients. Multivariate analysis showed that patients with PSC had a higher risk of death than those with other histological subtypes of NSCLC (HR: 1.34, *P* < 0.001). Furthermore, with 1:1 matching by propensity score, approximately 418 patients in both groups and PSC patients still had a higher risk of death than other types of NSCLC (HR:1.34, *P* < 0.001). A study from the Mayo Clinic arrived at a similar conclusion (30). Consistent with the above findings, our data showed that the median DFS and OS times in patients with operable PSC were 7.3 months and 12.0 months, respectively. It should be mentioned that in our study, the surgical modality was the only variable that affected OS in patients with PSC. This may be because patients who underwent pneumonectomy had a later pTNM stage than those who underwent lobectomy.

Recently, as more studies have been reported, ICIs have been shown to be advantageous in the management of these aggressive tumors (13, 31, 32). A study of ICIs in advanced PSC included five patients, all of whom had received ICI therapy, and the TPS of PD-L1 in all cases was >75%. Four of the five patients showed a response, including one with complete remission. During follow-up, one patient died of infectious complications after 23 months, with no signs of progression. Four patients continued to survive, with sustained survival between 14.0-33.0 months (33). Domblides et al. (14) reported outstanding clinical efficacy of ICIs in a total of 37 patients receiving immunotherapy. A study with the largest sample size to date also confirmed that ICI treatment offers promising results for patients with PSC (34). Therefore, the immunophenotyping in our present study to guide immunotherapy in PSC has more practical clinical implications.

To explore the reasons for the high response rate of PSC to ICIs, we performed PD-L1 expression in the intratumoral region of 32 patients with PSC. Up to 59.4% of the patients were identified as PD-L1-positive (TPS>1%), and 34.4% of the patients had a TPS score >50%, which might explain the advantages of immunotherapy in PSC. Consistent with our data, Velcheti et al. showed that 9 of 13 patients with PSC (69.2%) were PD-L1-positive with higher levels than in the common type of NSCLC (35). Subsequently, a series of studies have confirmed that, compared with other types of NSCLC, PSC has a higher positive rate of PD-L1 (15, 36). Additionally, we analyzed the predictive role of PD-L1 expression in patient prognosis. Although not statistically different, our data support the hypothesis that PD-L1-positive patients have poor survival rates. Previous studies corroborate our findings (15, 37).

However, in addition to PD-L1 expression in tumor cells, CD8+ T cell infiltration in the TME can also be used as a biomarker for predicting the efficacy of immunotherapy in NSCLC (38, 39). Based on this, some scholars have attempted to study the relationship between CD8+ T cell infiltration and clinical outcomes in PSC. Zhang et al. (40) conducted a study on the immune microenvironment of PSCs. Both PD-L1 and CD8+ T cell infiltration were observed in this study. Their data showed that, among 38 PSC patients, PD-L1-positive and CD8+ T-cell infiltrated patients accounted for 55.3% and 73.7%, respectively. Further analysis showed that PD-L1 expression was positively correlated with CD8+ T cell infiltration (*P* < 0.01). Likewise, our results confirmed the above; among the 32 patients, 21 (65.6%) were identified as positive for CD8+ T cell infiltration in intratumoral region. In addition, we also detected CD8+ T cells in the paired peritumoral regions because the infiltration of CD8+ T cells in different spatial regions can be more comprehensive for immunophenotyping of PSC. Previous studies have confirmed that, according to this model, tumors can be divided into three types: immune-inflamed, immune-excluded, and immune desert (21). It is worth noting that the immune-inflamed type is also called “hot tumor”, which is sensitive to immunotherapy (41, 42). Based on the infiltration status of CD8+ T cells in different spatial regions, we performed immunophenotyping of patients, of these 32 participants, 65.6% (21/32), 15.6% (5/32), and 18.8% (6/32) were classified as immune-inflamed, immune-excluded, and immune-desert, respectively.

Immunophenotyping based on CD8+ T-cell infiltration does not reflect PD-L1 expression in tumor cells, which is considered an objective criterion for the prediction of immunotherapy efficacy. Based on this, immune tetratyping that integrates CD8+ T cell infiltration and tumor cell PD-L1 expression has been favored by researchers (23, 43, 44). Researchers have applied this type of immune typing to the study of tracheal tumors and found that 60.0% of tracheal squamous cell carcinomas are type I (PD-L1+/CD8+, adaptive immune resistance). In tracheal adenoid cystic carcinoma, half are type II (PD-L1-/CD8-, immunologic ignorance) or type IV (PD-L1-/CD8+, tolerance) (20). In our study, nearly half of the patients with PSC were classified as having type I (PD-L1+/CD8+, adaptive immune resistance), suggesting that this population may benefit from single-agent ICIs. For patients with immune types II (PD-L1-/CD8-, immunological ignorance) and III (PD-L1+/CD8-, intrinsic induction), because of a lack of CD8+ T cell infiltration, PD-L1/PD-1 inhibitors alone will not work, and a combined anti-PD-1/PD-L1 therapy strategy that promotes CD8+ T cell infiltration should be recommended. Finally, for type IV (PD-L1-/CD8+, tolerated) patients, other immunotherapy modalities should be considered.

Our study has the following limitations. First, although we included a 10-year period with patients from two centers, the sample size of the study still did not allow for further stratified analysis. Second, due to the low incidence of PB, there were no such patients in our study, therefore, we could not represent PSC in a strict sense. Finally, the time span of the enrolled patients was large, and the diversity of their treatment regimens affected the prognostic analysis.

In conclusion, we have comprehensively and systematically explored the TME characteristics of PSC and their subtypes. PD-L1 is highly expressed in tumor cells, and CD8+ T cells are significantly increased in the TME. The majority of PSC patients were classified as immune-inflamed type and type I (PD-L1+/CD8+, adaptive immune resistance), suggesting that these special types of NSCLC patients may benefit more from immunotherapy.

## Data availability statement

The original contributions presented in the study are included in the article/supplementary material. Further inquiries can be directed to the corresponding authors.

## Ethics statement

The studies involving human participants were reviewed and approved by ethics committee of Shaanxi Provincial People’s Hospital (approval number:20220621). The patients/participants provided their written informed consent to participate in this study. Written informed consent was obtained from the individual(s) for the publication of any potentially identifiable images or data included in this article.

## Author contributions

JZ, TJ, and YM participated in study design and study conception. JZ and YM performed statistical analysis. HW, JZ, and TJ performed the surgery. YM, WL, and ZL performed pathological diagnosis. JZ and YM drafted the manuscript. All authors provided critical review of the manuscript and approved the final draft for publication.

## Funding

This study was supported by grants from the Wu Jieping Medical Foundation (320.6750.17527), and Special Fund for Elite Talents of Shaanxi Provincial People’s Hospital(2021JY-20).

## Conflict of interest

The authors declare that the research was conducted in the absence of any commercial or financial relationships that could be construed as a potential conflict of interest.

## Publisher’s note

All claims expressed in this article are solely those of the authors and do not necessarily represent those of their affiliated organizations, or those of the publisher, the editors and the reviewers. Any product that may be evaluated in this article, or claim that may be made by its manufacturer, is not guaranteed or endorsed by the publisher.
